# 

**Published:** 1970-07

**Authors:** 


RECEIVED
JUL 15 1970
National Library
of Medicine

### BRISTOL MEDICO-CHIRURGICAL JOURNAL

NATIONAL LIBRARY OF MEDICINE

INDEX COPY

RECD. JUL 16 1970

INDEX DEC 31 1970

INPUT

